# Antiestrogen Resistant Cell Lines Expressing Estrogen Receptor α Mutations Upregulate the Unfolded Protein Response and are Killed by BHPI

**DOI:** 10.1038/srep34753

**Published:** 2016-10-07

**Authors:** Chengjian Mao, Mara Livezey, Ji Eun Kim, David J. Shapiro

**Affiliations:** 1 Department of Biochemistry, University of Illinois at Urbana-Champaign, Urbana, IL 61801, USA.

## Abstract

Outgrowth of metastases expressing ERα mutations Y537S and D538G is common after endocrine therapy for estrogen receptor α (ERα) positive breast cancer. The effect of replacing wild type ERα in breast cancer cells with these mutations was unclear. We used the CRISPR-Cas9 genome editing system and homology directed repair to isolate and characterize 14 T47D cell lines in which ERαY537S or ERαD538G replace one or both wild-type ERα genes. In 2-dimensional, and in quantitative anchorage-independent 3-dimensional cell culture, ERαY537S and ERαD538G cells exhibited estrogen-independent growth. A progestin further increased their already substantial proliferation in micromolar 4-hydroxytamoxifen and fulvestrant/ICI 182,780 (ICI). Our recently described ERα biomodulator, BHPI, which hyperactivates the unfolded protein response (UPR), completely blocked proliferation. In ERαY537S and ERαD538G cells, estrogen-ERα target genes were constitutively active and partially antiestrogen resistant. The UPR marker sp-XBP1 was constitutively activated in ERαY537S cells and further induced by progesterone in both cell lines. UPR-regulated genes associated with tamoxifen resistance, including the oncogenic chaperone BiP/GRP78, were upregulated. ICI displayed a greater than 2 fold reduction in its ability to induce ERαY537S and ERαD538G degradation. Progestins, UPR activation and perhaps reduced ICI-stimulated ERα degradation likely contribute to antiestrogen resistance seen in ERαY537S and ERαD538G cells.

Endocrine therapy for estrogen receptor α (ERα) positive breast cancers employs aromatase inhibitors to block estrogen production and tamoxifen and fulvestrant/Faslodex/ICI 182,780 (ICI) that compete with estrogens for binding to ERα. For advanced metastatic breast cancer, selection and outgrowth of tumors resistant to endocrine therapy and expressing ERα mutations ERαY537S and ERαD538G is common^1^,^2^,^3^,^4^. There is compelling evidence these mutations are resistant to aromatase inhibitors^1^,^2^,^3^,^4^,^5^,^6^,^7^. While most evidence suggests they are also clinically resistant to tamoxifen and fulvestrant/ICI^4^,^8^,^9^, recent studies demonstrated increased prevalence of ERα mutations in breast cancers of patients treated with aromatase inhibitors, but not in patients treated with fulvestrant^5^, or tamoxifen^6^. These researchers question the association of ERα mutations with clinical resistance to fulvestrant and tamoxifen. In studies mostly using transfected ERα negative cells, the mutants were reported to be resistant to tamoxifen and ICI^4^, resistant to tamoxifen but sensitive to ICI^3^ and sensitive to antiestrogen inhibition^2^,^10^. Previously described systems for analyzing the ERαY537S and ERαD538G mutations were not ideal. Cell lines derived from circulating tumor cells exhibit multiple genetic changes and lack a control cell line. Transfected ERα negative cell lines do not exhibit estrogen-ERα regulated proliferation and display a different ERα-regulated gene expression pattern than ERα positive breast cancer cells^11^. A better experimental model would compare cells expressing the ERα mutations and wild type ERα in a defined genetic background in an ERα positive breast cancer cell whose proliferation is stimulated by estrogen. We therefore used the CRISPR-Cas9 gene editing system to produce multiple cell lines in which one or both copies of the wild type ERα gene was replaced by ERαY537S or ERαD538G.

Although the most common application of the CRISPR-Cas9 system is targeted gene inactivation by non homologous end joining (NHEJ) to repair the Cas9 generated DNA break, when a homologous repair donor is present, a homology-directed repair process (HDR) can precisely insert a sequence containing the desired modification into the gene of interest. Because the frequency of HDR is usually extremely low^12^,^13^,^14^,^15^,^16^, the CRISPR-Cas9 system has rarely been used to successfully repair or insert specific mutations in both copies of endogenous genes in a cancer cell line. We used the CRISPR-Cas9 gene editing system to generate 50 clonal cell lines with one or both copies of endogenous wild-type ERα replaced with ERαY537S or ERαD538G.

Although progesterone reportedly plays a role in breast cancer progression^17^,^18^, a recent study concluded that when E_2_ is present, progesterone enhances tamoxifen’s effectiveness as an antiestrogen^19^. The effect of progestins in cells expressing ERα mutations had not been explored.

We showed that the estrogen, 17β-estradiol (E_2_), acts through ERα to elicit extremely rapid and functionally important anticipatory activation of the endoplasmic reticulum stress sensor, the unfolded protein response (UPR)^20^. Moreover, activation of a UPR gene index at diagnosis is a powerful prognostic indicator, tightly correlated with subsequent resistance to tamoxifen therapy^20^. This ERα-regulated UPR pathway is targeted by BHPI, our recently described noncompetitive ERα biomodulator. BHPI hyperactivates the UPR, converting it from cytoprotective to cytotoxic^21^,^22^. While BHPI is effective in tamoxifen-resistant breast cancer cells expressing wild type ERα, its effectiveness in cells expressing ERα mutations associated with metastases was unknown.

Here we describe the effects of OHT, ICI and BHPI on proliferation of the ERαY537S and ERαD538G cells in anchorage dependent and anchorage independent culture with and without a progestin, analyze gene expression, and evaluate estrogen-independent and progestin-stimulated UPR activation and reduced ERα degradation as potential contributors to antiestrogen resistance.

## Results

### Using CRISPR-Cas9 to replace wild-type ERα with ERαY537S or ERαD538G

Our strategy is illustrated in Supplementary Fig. S1. To increase the frequency of HDR, we used 2 guide sequences. Additionally, to abolish repeated cutting of homology-replaced sequences by Cas9, we changed one nucleotide in both PAM sequences of the HDR template without altering the ERα amino acid sequence. To facilitate identification of cells in which the mutant ERα fragment replaced the wild-type ERα fragment, we inserted AclI and SpeI sites into the HDR template.

We chose T47D cells because they contain 2 copies of the ERα gene and exhibit E_2_ dependent growth^23^. Previous transfection studies suggested ERαY537S and ERαD538G cells grow without E_2_^1^,^2^,^3^. We exploited this phenotype during outgrowth and selection of colonies of ERαY537S and ERαD538G cells.

Individual colonies were pulled, grown out and genotyped. Genomic DNA spanning the HDR template was amplified and analyzed by digestion with AclI or SpeI. Replacement of wild-type ERα results in two bands due to AclI or SpeI cutting (Supplementary Fig. S1). 50 of 65 clonal cell lines contained single or double gene replacements (Supplementary Fig. S2). Expression of the ERα mutations in mRNA was confirmed by synthesizing and digesting cDNA. The presence of the mutated ERα sequences in the cell lines was verified by DNA sequencing (Supplementary Fig. S1). We used ERαY537S-4 because its properties were typical of the ERαY537S cell lines and because mapping and sequencing confirmed 2 distinct and independent gene replacement events (Supplementary Fig. S1 legend); ERαD538G-1 was chosen as typical of the ERαD538G cell lines. Sequencing and Western blotting with N-terminal ERα antibody showed that NHEJ introduced diverse indels in the non-replaced copy of ERα (Supplementary Figs 1 and 2). We therefore focused primarily on the double replacement cell lines.

Western blotting to compare ERα levels in parental T47D cells to levels of ERαY537S and ERαD538G in single and double replacement cell lines showed some heterogeneity in expression level of ERαY537S and ERαD538G. The ERα mutations were expressed at levels similar to or lower than wild-type ERα (Supplementary Fig. S2).

### ERαY537S and ERαD538G cells exhibit E_2_-independent growth and partial resistance to OHT and ICI

**Figure 1:**
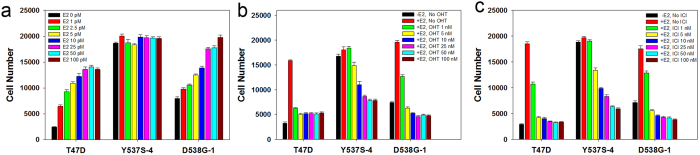
In standard anchorage-dependent 2D culture, ERαY537S-4 and ERαD538G-1 cells exhibit E_2_-independent proliferation and partial resistance to OHT and ICI. (**a**) Dose-response study comparing the effects of increasing concentrations of E_2_ on the proliferation of T47D, ERαY537S-4 and ERαD538G-1 cells. (**b**,**c**) Dose-response studies comparing the effects of increasing concentrations of OHT (**b**) and ICI (**c**) on the proliferation of T47D, ERαY537S-4 and ERαD538G-1 cells. Data is mean ± SEM (n = 8).

Although the clonal cell lines were isolated and maintained without E_2_, it was unknown whether E_2_ further stimulates their growth. As expected, the parental T47D cells exhibited little growth without E_2_ and dose-dependent increases in growth with E_2_ (Fig. 1a)^21^. The ERαY537S-4 cells and the other ERαY537S cell lines were completely E_2_-independent for growth. E_2_ further stimulated the more modest E_2_-independent proliferation of ERαD538G-1 and other ERαD538G cells (Fig. 1a and Supplementary Fig. 3).

We next performed dose response studies evaluating the effect of OHT and ICI on proliferation of the T47D, ERαY537S and ERαD538G cell lines. To facilitate comparisons, T47D and mutant cells were maintained in medium containing E_2_. In T47D cells, OHT and ICI nearly abolished proliferation at a 50–1,000 fold molar excess over E_2_ (Fig. 1b,c). In contrast, the mutant cell lines, especially ERαY537S-4, exhibited some resistance (Fig. 1b,c; blue bars). Additionally, while T47D cells displayed negligible growth in ICI, all seven ERαY537S and ERαD538G double replacement cell lines displayed at least some growth in ICI (Supplementary Fig. S3). In longer 8-day cultures, compared to T47D cells, the mutant cells displayed increased proliferation in OHT and ICI (Supplementary Fig. S4).

### BHPI blocks growth of ERαY537S and ERαD538G cells

**Figure 2:**
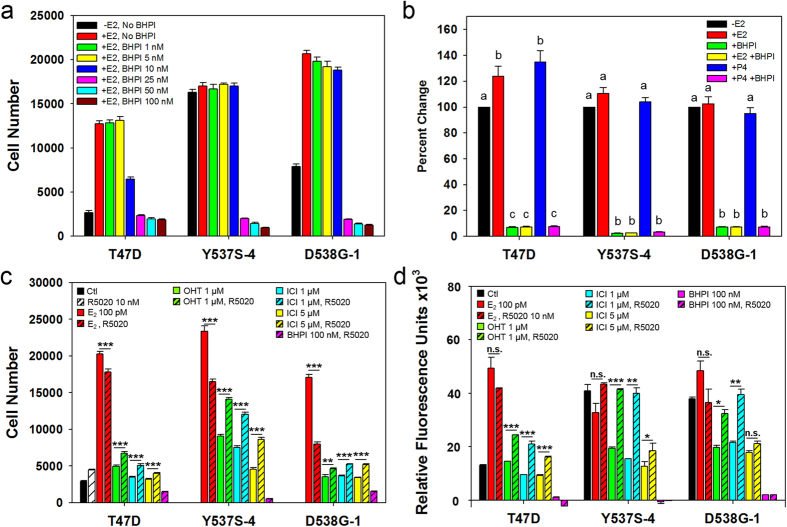
ERαY537S-4 and ERαD538G-1 cells show antiestrogen resistant growth in 2D and 3D culture, but are killed by BHPI. (**a**) Dose-response study of the effect of BHPI on the proliferation of T47D, ERαY537S-4 and ERαD538G-1 cells in standard anchorage-dependent 2D culture. (**b**) BHPI inhibits protein synthesis >90% in T47D, ERαY537S-4 and ERαD538G-1 cells. Cells were treated with vehicle, 1 nM E_2_ or 1 nM P_4_ with or without 100 nM BHPI for 3 hr. Data is mean ± SEM (n = 5). (**c**) In 2D culture, R5020 reduces the ability of OHT and ICI to inhibit proliferation of ERαY537S-4 and ERαD538G-1 cells. (**d**) In 3D culture, ERαY537S-4 and ERαD538G-1 cells are highly antiestrogen resistant and resistance is robustly increased by R5020, but the cells are killed by BHPI. Conditions (**a**,**c**,**d**) as noted, with 100 pM E_2_ unless otherwise stated. Data (**a**,**c**,**d**) is mean ± SEM (n = 8). For (**b**) letters indicate a significant difference among groups (p < 0.05) using one-way ANOVA followed by Tukey’s post hoc test within each cell line. -E2 in each cell line set to 100%. For (**c**,**d**) *p < 0.05, **p < 0.01, ***p < 0.001 (by Student’s T test) comparing treatment to treatment + R5020.

Since the ERαY537S and ERαD538G cell lines displayed partial resistance to OHT and ICI, we evaluated the ability of our recently described noncompetitive ERα biomodulator, BHPI, to inhibit proliferation of ERαY537S and ERαD538G cells^21^. At 25 nM, BHPI completely inhibited proliferation of T47D, ERαY537S-4 and ERαD538G-1 cells and the 14 characterized ERα mutant cell lines (Fig. 2a and Supplementary Fig. S5). In longer 8-day cultures, BHPI continued to completely block proliferation of T47D, ERαY537S-4 and ERαD538G-1 cells (Supplementary Fig. S4).

### BHPI potently inhibits protein synthesis in ERαY537S and ERαD538G cells

BHPI acts through ERα to induce toxic hyperactivation of all three arms of the unfolded protein response (UPR)^21^,^22^,^24^. By hyperactivating the protein synthesis inhibiting PERK arm of the UPR, BHPI induces near quantitative inhibition of protein synthesis^21^. Potent long-term inhibition of protein synthesis is central to BHPI’s ability to block growth and kill ERα positive breast cancer cells^21^. While the hormones E_2_ and progesterone (P_4_) had minimal effects on protein synthesis, BHPI (100 nM) rapidly inhibited protein synthesis by >90% in T47D, ERαY537S-4 and ERαD538G-1 cells (Fig. 2b). Thus, the ERαY537S and ERαD538G mutations have no effect on BHPI’s ability to block cell proliferation and strongly inhibit protein synthesis by hyperactivating the UPR.

### ERαY537S and ERαD538G cells exhibit progesterone-stimulated antiestrogen-resistant, BHPI-sensitive, growth in anchorage-independent culture

Although anchorage-independent growth in 3-dimensional (3D) culture is a hallmark of cancer, it has been difficult to study quantitatively. Modifying earlier methods^25^, we developed a protocol for quantitative assessment of cell proliferation in a more biologically relevant anchorage-independent 3D culture in soft agar.

We compared the effects of OHT (1 μM; 10,000 fold excess over E_2_), ICI (1 and 5 μM), BHPI (100 nM) and the synthetic progestin R5020 (10 nM), on proliferation of ERαY537S-4 and ERαD538G-1 cells in anchorage-dependent 2D and anchorage-independent 3D culture. 5 μM OHT was not used because it exhibits nonspecific toxicity^26^,^27^,^28^. In standard 2D culture, the ERαY537S-4 cells exhibited reduced, but significant proliferation in OHT and ICI, which was increased by R5020. The ERαD538G-1 cells exhibited little proliferation in OHT and ICI, which increased slightly in R5020 (Fig. 2c, compare open bars: no R5020, hatched bars: +R5020).

In quantitative 3D culture, ICI was more effective than OHT in blocking growth of T47D cells. Impressively, proliferation of the mutants in 1 μM OHT and ICI was >50% of control, a dramatic increase compared to 2D culture (Fig. 2d). At 1 μM, ICI was no more effective than OHT in inhibiting proliferation of the mutants. Notably, in 3D culture, ERαY537S-4 and ERαD538G-1 cells grown in R5020 were completely resistant to growth inhibition by 1 μM OHT and ICI (Fig. 2d).

In both 2D and 3D culture, BHPI stopped growth and killed the ERαY537S-4 and ERαD538G-1 cells grown with or without R5020 (Fig. 2a,c,d). Neither growth in a progestin, nor growth in 3D culture, impaired BHPI’s impressive ability to block growth and kill ERαY537S-4 and ERαD538G-1 cells.

### E_2_-ERα regulated genes are constitutively expressed and antiestrogen resistant in ERαY537S and ERαD538G cells

**Figure 3:**
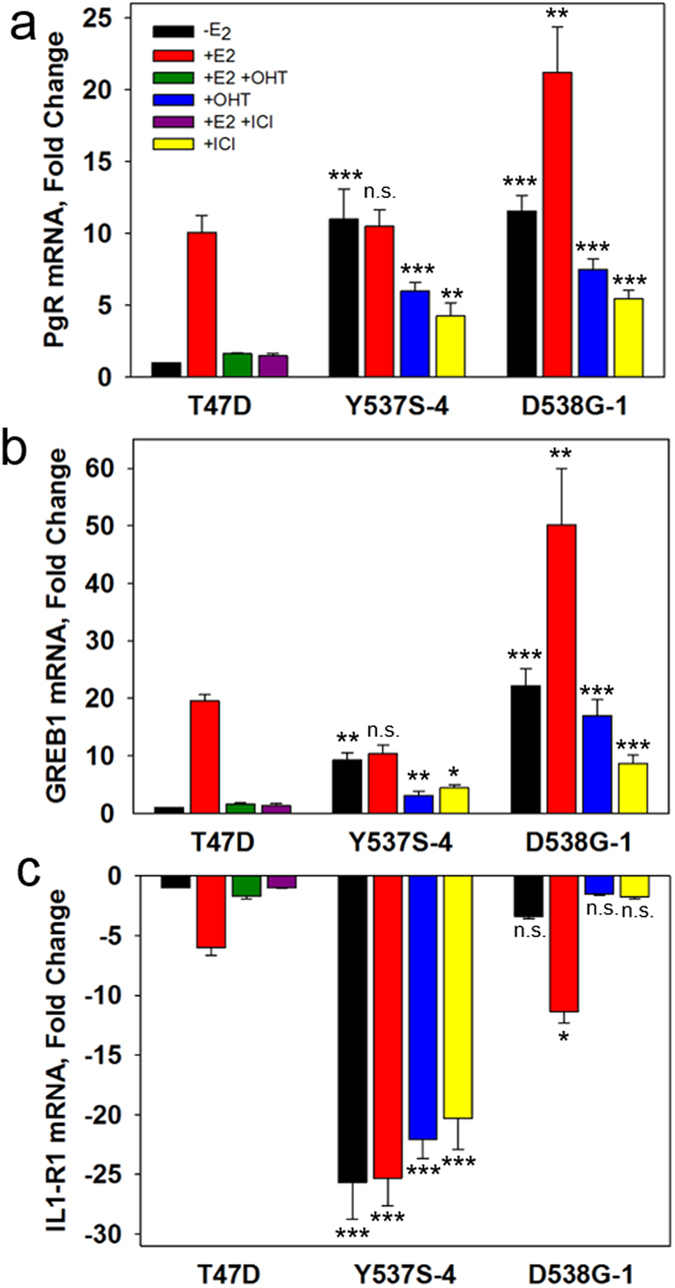
ERαY537S-4 and ERαD538G-1 cells exhibit constitutive, E_2_ independent, gene expression with significant resistance to inhibition by OHT and ICI. (**a**) Levels of PgR mRNA or (**b**) GREB1 mRNA after 4 hr treatment and (**c**) IL1-R1 mRNA after 24 hr treatment in T47D, ERαY537S-4 and ERαD538G-1 cells. *p < 0.05, **p < 0.01, ***p < 0.001, for each treatment, comparing mutant cell lines to wild type T47D using one-way ANOVA followed by Dunnett’s post hoc test. E_2_ 1 nM; OHT 1 μM; ICI 1 μM; Data is mean ± SEM (n = 3); qRT-PCR; -E_2_ in parental T47D cells set to 1 in (**a,b**) and −1 in (**c**).

To explore factors that might contribute to antiestrogen resistance in the ERαY537S and ERαD538G mutants, we evaluated the effects of E_2_, OHT and ICI on ERα-regulated transcription. Our endogenous test genes were progesterone receptor (PgR), the oncogenic co-regulator, GREB1, and the down-regulated gene, IL1-R1. In parental T47D cells, E_2_ induction of PgR and GREB1 mRNAs and down regulation of IL1-R1 mRNA was abolished by OHT and ICI (Fig. 3a–c). Consistent with cell proliferation data (Fig. 1a), induction and repression of gene expression by ERαY537S was estrogen independent, but was only partially constitutive for ERαD538G (Fig. 3a–c). Induction of PgR and GREB1 mRNAs was partially OHT and ICI resistant in both ERαY537S-4 and ERαD538G-1 cells (Fig. 3a,b). In contrast, down-regulation of IL1-R1 was highly resistant to OHT and ICI in ERαY537S-4 cells (Fig. 3c). Perhaps because IL1-R1 down-regulation was mostly estrogen dependent in ERαD538G cells, it was highly sensitive to inhibition by OHT and ICI (Fig. 3c).

In the ERαY537S-5 and ERαD538G-4 cell lines, in which one copy of the ERα gene was replaced with a mutant ERα and the other contained an indel resulting in reading frame shift and truncated ERα, induction of PgR and GREB1 and down-regulation of IL1-R1 was estrogen independent and was much reduced compared to the double replacement cell lines. The modest induction and repression of gene expression in ERαY537S-5 and ERαD538G-4 cells was highly resistant to inhibition by OHT and ICI (Supplementary Fig. 6).

### ICI 182,780 exhibits a reduced ability to induce degradation of ERαY537S and ERαD538G

**Figure 4:**
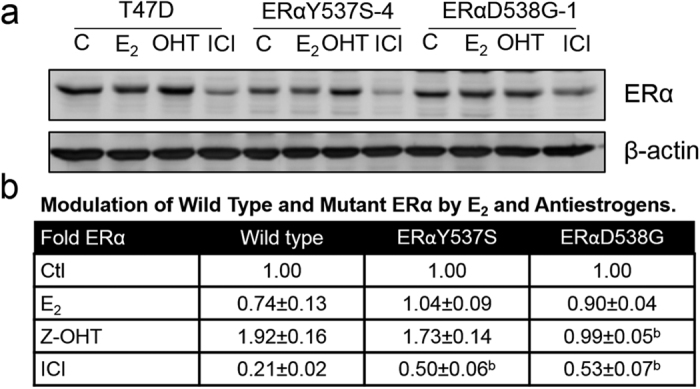
ERαY537S and ERαD538G exhibit altered ligand-dependent degradation. (**a**) Western blot comparing the effects of E_2_, OHT and ICI on levels of ERα in T47D, ERαY537S-4 and ERαD538G-1 cells. Cells were treated with vehicle (C) or 10 nM E_2_ (18 hr), or with 1 μM OHT or 1 μM ICI (24 hr). (**b**) Quantitation of densitometry of ERα levels in ligand-treated T47D, ERαY537S and ERαD538G double replacement cell lines, within each cell line treatments were compared to vehicle control. Data is mean ± SEM (ERαY537S: n = 4 cell lines; ERαD538G: n = 3 cell lines; T47D n = 3 experiments); (**b**) p < 0.01 (by Student’s T test), for each treatment, comparing mutant to wild-type ERα in T47D cells.

ICI induces ERα degradation. Since the ERαY537S-4 and ERαD538G-1 cells were ICI resistant in 3D culture, we examined the effect of ICI and other ligands on levels of ERαY537S and ERαD538G. In the presence of E_2_, wild-type ERα exhibited modest down-regulation, which was less evident in the mutants. OHT is a weak agonist and stabilizes ERα. OHT increased levels of ERαY537S but had little effect on the level of ERαD538G. There was a highly significant >2 fold reduction in the ability of ICI to induce degradation of both the double and single replacement ERαY537S and ERαD538G mutants (Fig. 4a,b and Supplementary Fig. S7).

### Markers of UPR activation are elevated in ERαY537S and ERαD538G cells

**Figure 5:**
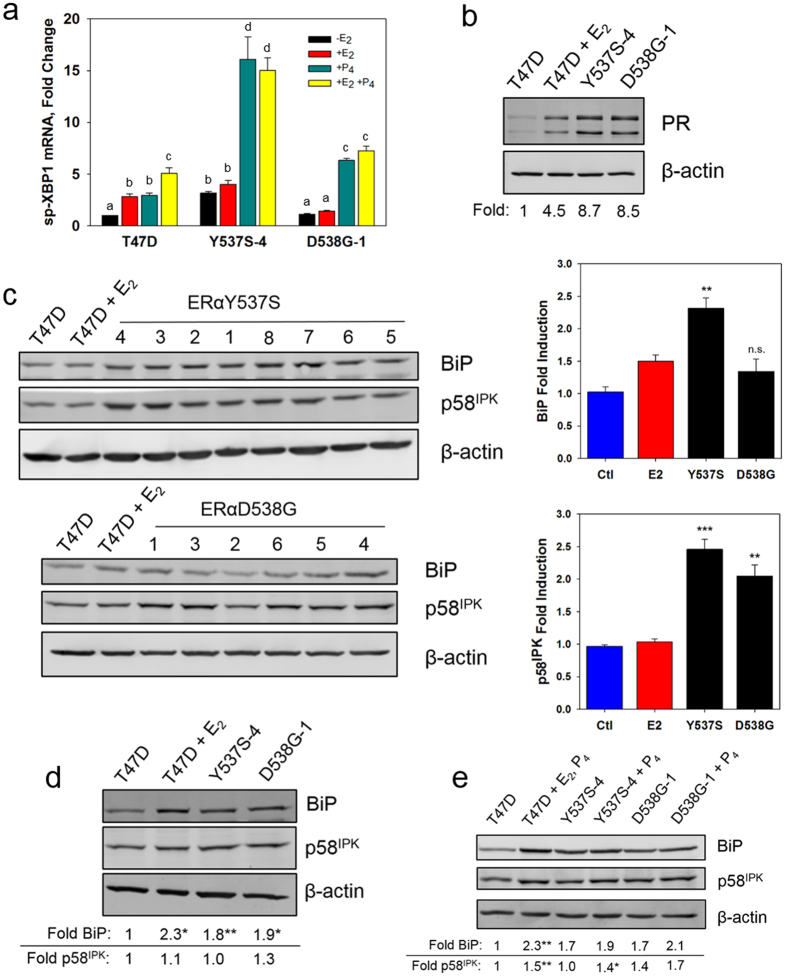
The UPR is activated in ERαY537S-4 and ERαD538G-1 cells and is further activated by progesterone. (**a**) sp-XBP1 mRNA is upregulated in ERαY537S-4 cells and P_4_ causes further upregulation in both cell lines. Letters indicate a significant difference among groups (p < 0.05) using one-way ANOVA followed by Tukey’s post hoc test. Cells were treated with vehicle, 1 nM E_2_ and/or 1 nM P_4_ for 4 hr. Data is mean ± SEM (n = 3); qRT-PCR; -E_2_ in T47D cells set to 1. (**b**) Progesterone receptor is highly upregulated in ERαY537S-4 and ERαD538G-1 cells. (**c**) Western blots (left) and quantitation (right) showing BiP and p58^IPK^ induction in all ERαY537S and ERαD538G cell lines. (**d**) Western blot showing induction of BiP and p58^IPK^ in ERαY537S-4 and ERαD538G-1 cells. (**e**) BiP is strongly induced and p58^IPK^ is induced by P_4_ in T47D cells, p58^IPK^ but not BiP is induced by P_4_ in ERαY537S-4 cells and neither is significantly induced by P_4_ in ERαD538G-1 cells. For (**b–d**) T47D cells were treated with vehicle or 10 nM E_2_ for 24 hr, mutant cell lines were treated with vehicle. In (**e**) T47D cells were treated with vehicle for 48 hr or with E_2_ for 24 hr to induce PR then P_4_ for 24 hr; ERαY537S-4 and ERαD538G-1 cells were treated with vehicle or P_4_ for 24 hr. Data is mean ± SEM. In (**c**) ERαY537S: n = 8 cell lines; ERαD538G: n = 6 cell lines; T47D n = 3 experiments. In (**d,e**) quantitation shown below is from the average of three identical blots. For (**c,d,e**) *p < 0.05, **p < 0.01, ***p < 0.001 (by Student’s T test), for each treatment, comparing each mutant to wild-type vehicle control (**c**,**d**) or comparing vehicle treated to P_4_ within each cell line (**e**).

E_2_ elicits a weak anticipatory activation of the UPR that is a strong prognostic marker correlated with tamoxifen resistance^20^,^24^,^29^. The effect of P_4_ on the UPR had not been explored. We therefore investigated the effect of estrogen and progesterone on UPR markers in the tamoxifen-resistant ERαY537S-4 and ERαD538G-1 cells. Activation of IRE1α, resulting in cleavage of XBP1 mRNA to spliced XBP1 (sp-XBP1), is a widely used marker of UPR activation. Activation of the UPR’s ATF6α and IRE1α arms induces the strongly oncogenic chaperone BiP/GRP78/HSPA5 by 1.5-3 fold^30^,^31^. Activation of these UPR arms also induces the anti-apoptotic protein p58^IPK^, the angiogenesis-related protein ERO1a, and the oncogenic UPR-related protein SERP1^32^,^33^,^34^. In T47D cells, E_2_ modestly induced sp-XBP1 mRNA (Fig. 5a)^20^. In the ERαY537S-4 cells sp-XBP1 levels were constitutively elevated and were not further increased by E_2_. In the ERαD538G-1 cells sp-XBP1 levels were minimally higher than basal levels and were not increased by E_2_ (Fig. 5a). UPR-induced BiP protein and ERO1a and SERP1 mRNAs were all constitutively elevated in the ERαY537S-4 cells and BiP and SERP1 were elevated in the ERαD538G-1 cells (Fig. 5c,d and Supplementary Fig. S8). Pooling data from all 14 mutant cell lines, BiP was significantly elevated in ERαY537S cells and p58^IPK^ was elevated in both ERαY537S and ERαD538G cells (Fig. 5c).

In T47D cells, E_2_-induced sp-XBP1 levels were further increased by P_4_. In the ERαY537S-4 and ERαD538G-1 cells, P_4_ elicited a strong E_2_-independent induction of sp-XBP1 mRNA (Fig. 5a). Consistent with the larger effect of progesterone on sp-XBP1 levels in the ERαY537S-4 and ERαD538G-1 cells (Fig. 5a), and with the constitutive induction of PgR mRNA (Fig. 3a), progesterone receptor (PR) was constitutively elevated to levels higher than in E_2_-treated T47D cells (Fig. 5b). P_4_ significantly induced levels of BiP and p58^IPK^ in T47D cells and further increased p58^IPK^ in ERαY537S-4 cells, but neither BiP nor p58^IPK^ were significantly further induced in ERαD538G-1 cells (Fig. 5e). Thus, progesterone further increases expression of some, but not all, UPR markers.

## Discussion

While the CRISPR-Cas9 genome editing system has been widely used to inactivate genes^35^,^36^,^37^,^38^,^39^,^40^, gene replacement studies often use a few cell lines exhibiting unusually high HDR frequency^14^,^15^,^16^,^41^. While our use of 2 guide sequences to increase the frequency of HDR may increase the possibility of off-target events^42^,^43^,^44^, our ability to confirm key observations in 4 ERαY537S and 3 ERαD538G clonal cell lines strongly suggests that the observed properties are due to ERα gene replacement. Because indirect evidence suggests long templates might increase HDR^45^, we used double-stranded templates with 1.2 kb arms, not the short ~50 bp single stranded arms usually used in HDR^46^.

Consistent with the frequency of error-prone NHEJ being much higher than the frequency of HDR^12^,^13^,^14^,^15^,^16^, all sequenced single replacement clones contained indels in the non-HDR ERα copy. Since ERα in these clones may exist in heterodimers, in which the indel-containing ERα monomer is non-functional, we focused on the double replacement cell lines. Notably, ~30% of cell lines replaced both copies of ERα. This suggests each wild-type ERα copy may undergo multiple NHEJ events before an indel destroys the guide-matched DNA region or a rare HDR event inactivates the PAM sequence.

This work exploited our ability to select for multiple clones expressing ERα mutants that grew out in the absence of E_2_. Based on calculations from our large sample size, it seems feasible, but very challenging, to replace genes in typical breast cancer cell lines without a selection. Recent studies, largely in HEK293 cells, describe methods for increasing the probability of HDR using inhibitors of NHEJ or silencing key molecules involved in NHEJ and optimized HDR templates that target the asymmetric mechanism of Cas9^14^,^15^,^16^.

Our data indicates that the ERαY537S and ERαD538G mutations are sufficient to convert an ERα positive cancer cell, exhibiting E_2_-dependent proliferation and growth inhibition by antiestrogens, to one exhibiting E_2_-independent proliferation and resistance to high concentrations of OHT and ICI. Importantly, since these experiments were the initial exposure of the ERαY537S and ERαD538G cells to antiestrogens, there was no prior selection for other changes favoring antiestrogen resistance. Consistent with earlier work, E_2_-independent proliferation will make tumors expressing these mutations resistant to aromatase inhibitors.

In more biologically relevant anchorage-independent 3D culture, the ERαY537S and ERαD538G cell lines exhibited robust proliferation in the presence of 1 μM z-OHT and 1 μM fulvestrant/faslodex/ICI 182,780 (Fig. 2d). Notably, the mutant cell lines continued to proliferate in 5 μM ICI. Since serum ICI in patients is 20–30 nM^47^,^48^, it is unlikely tumors can concentrate ICI to levels that exceed the 1 and 5 μM in which the mutant cell lines proliferate in 3D culture.

It is widely accepted that ICI is a more nearly pure antagonist than OHT. Since OHT stabilizes and ICI induces degradation of ERα, the ~9 fold lower level of ERα in ICI-treated compared to OHT-treated cells likely makes an important contribution to the increased effectiveness of ICI in T47D cells. However, in the ERαY537S and ERαD538G cell lines, ICI was no more effective in blocking cell growth than OHT, and ICI exhibited a greater than 2 fold reduced ability to induce degradation of the mutant ERαs. Instead of the ~9 fold higher level of ERα in OHT-treated versus ICI-treated T47D cells, the difference in mutant ERα levels was reduced to 2–3 fold. The diminished ability of ICI to induce degradation of the ERα mutants likely contributes to ICI resistance of ERαY537S and ERαD538G cells. Notably, the ERαY537N mutation is also resistant to ICI-induced degradation^49^. However, in a very recent report, ERαD538G in one patient’s circulating tumor cells was not resistant to degradation^50^. This suggests that ICI-induced ERα degradation may also depend on subtle genetic differences in tumors. Of note, using double replacement cell lines may facilitate degradation studies; using current antibodies it is not possible to distinguish between wild type ERα and the mutant ERαs containing these single amino acid changes.

OHT is an antagonist because it induces a different conformational change in ERα than E_2_. Very recently, using structural, computational and biophysical approaches Greene and coworkers suggested that the ERαY537S and ERαD538G mutations confer partial antiestrogen resistance because they exhibit reduced affinity for OHT and because they induce a constitutively active conformation that resists deformation by OHT. Moreover, they suggested that ICI, which disorders helix 12 of ERα, might be a therapeutically effective antagonist, blocking activity of ERαY537S and ERαD538G^51^. They also report that ERαY537S is in a conformation that exhibits higher constitutive binding of coactivators than ERαD538G^51^. Our experimental data on differences in proliferation and gene expression between the ERαY537S and ERαD538G cell lines is consistent with their proposed differences in coactivator interaction. Supporting the view that ERαY537S and ERαD538G are in a constitutively active conformation that resists antiestrogens, both gene expression in standard cell culture and proliferation in 3D culture are constitutive and largely resistant to saturating OHT and ICI. Elevated coactivator expression and a shift in coactivators, often including down-regulation of GREB1, have been linked to antiestrogen resistance^52^,^53^,^54^,^55^,^56^,^57^. In contrast, GREB1 is constitutively upregulated in the mutant cell lines, suggesting antiestrogen resistance is not caused by a shift away from GREB1.

In breast cancer cells expressing wild type ERα and PR, progesterone increases stemness and markers associated with therapy resistance^17^. Moreover, progesterone can be mitogenic and mediate cell survival in 3D cultures^17^. However, recent xenograft and explant studies show that when estrogen is present, progestins enhance sensitivity of breast cancers expressing wild type ERα to tamoxifen^19^. Consistent with an earlier study^58^, PR was constitutively expressed to extremely high levels in the ERαY537S and ERαD538G cells, suggesting it might play an important role. We report that a progestin decreases sensitivity of ERαY537S and ERαD538G cells to growth inhibition by OHT and ICI in both 2D and 3D culture. In 3D cultures treated with a progestin, 1 μM OHT and ICI have lost the ability to inhibit proliferation of ERαY537S and ERαD538G cells. Since progestin had little ability to reverse OHT and ICI inhibition of T47D cell growth, the progestin effect is likely related to the ERαY537S and ERαD538G mutations. Consistent with the possibility that serum progestin might influence antiestrogen resistance in women whose breast cancers express the ERαY537S and ERαD538G mutations, progesterone levels in post-menopausal women are 0.6–3 nM^59^,^60^,^61^. Moreover, breast cancer cells expressing both ERα and PR, or overexpressing PR alone, may be sensitive to very low concentrations of hormone^17^,^18^. Since the ERαY537S and ERαD538G cells constitutively express very high levels of PR, the low serum concentrations of progestins in postmenopausal women together with high PR could well have functional effects on antiestrogen resistance in metastatic breast cancers expressing ERα mutations.

Weak activation of the UPR is protective in cancer and is associated with a poor prognosis, while extensive and sustained UPR activation by BHPI is toxic^21^,^22^,^24^,^29^. Since we cannot currently analyze protein and mRNA levels in cells from 3D cultures in soft agar, the UPR studies were carried out in standard 2D cell culture. The ERαY537S cells exhibited constitutive activation of the IRE1α arm of the UPR, as indicated by estrogen-independent expression of sp-XBP1 and induction of the oncogenic chaperone BiP. In contrast, the ERαD538G cells exhibited low expression of sp-XBP1 and a much smaller induction of BiP. This is consistent with the ERαD538G mutant showing a lower level of constitutive growth and antiestrogen resistance in cell proliferation and in E_2_-ERα regulated gene expression. Since our 2D cell culture data shows that ERαY537S cells exhibit significant resistance to OHT and ICI and the ERαD538G cells exhibited very little resistance (Fig. 2c), this data is consistent with a link between constitutive UPR activation and antiestrogen resistance. Moreover, progesterone increased sp-XBP1 expression to a higher level in the ERαY537S cells (Fig. 5a) than in the ERαD538G cells. Compared to the ERαD538G cells, the ERαY537S cells exhibited increased progesterone-stimulated resistance to OHT and ICI (Fig. 2c). Some oncogenic UPR markers, notably SERP1 were elevated in the mutant cell lines, but others, such as p58^IPK^ and ERO1a behaved differently in different cell lines. Although progesterone strongly induced sp-XBP1, it did not significantly further increase the strongly elevated level of BiP in the mutant cell lines and only further induced p58^IPK^ in the ERαY537S cells. Thus, while constitutive UPR activation likely contributes to the antiestrogen resistant phenotype seen in the ERαY537S and ERαD538G cell lines, the extent to which it does is unclear, and effects are likely complex.

In 2D culture R5020 increased proliferation of the ERαY537S and ERαD538G cells in OHT and ICI; in 3D culture, R5020 abolished growth inhibition by OHT and ICI. This effect of progestin-PR may not only be mediated through further activation of the UPR but also through modulation of ERα action and PR’s own transcriptomic program^19^. Although further study is needed, progestins seem likely to enhance antiestrogen effectiveness in cancer cells expressing the ERαY537S and ERαD538G mutations.

Although the mutations exhibit an altered ERα conformation^5^, and are close to the proposed BHPI binding site^21^, BHPI was as effective in blocking proliferation of all 14 of the ERαY537S and ERαD538G cell lines as it was in T47D cells. Moreover, in 3D culture where the ERαY537S-4 and ERαD538G-1 cells exhibited robust proliferation in 1 μM OHT and ICI, 100 nM BHPI completely blocked proliferation and killed the cells. In cancer cells expressing wild-type ERα, BHPI strongly activates the PERK arm of the UPR, resulting in rapid, near-quantitative inhibition of protein synthesis^21^. In the parental T47D cells and in the ERαY537S-4 and ERαD538G-1 cells, 100 nM BHPI elicited >90% inhibition of protein synthesis. This supports BHPI acting in the ERα mutant cell lines by the same pathway we described in cancer cells expressing wild-type ERα^21^.

We used the CRISPR-Cas9 genome editing system to generate precise replacements of both copies of a steroid receptor gene. Breast cancer cell lines expressing these ERα mutations associated with metastatic breast cancer are highly resistant to endocrine therapy. Taken together, our data and the recent structural and biophysical studies suggest that a constitutively active ERα conformation, altered ICI-induced degradation of ERα, progestin and UPR activation may all contribute to the antiestrogen resistant phenotype. Notably, the preclinical drug candidate BHPI retains its effectiveness against these resistant breast cancer cells.

## Methods

### Cell culture, reagents and western blotting

Cell culture medium and conditions were as previously described^62^. T47D cells were from ATCC (VA). z-OHT was from Sigma-Aldrich (MO) and ICI 182,780 was from Tocris Biosciences (Bristol, UK). Additional details are in Supplementary Methods.

### Generation of ERαY537S and ERαD538G cell lines

Our protocol was loosely based on previous work^46^. Two guide sequences (Supplementary Fig. S1) were cloned into pSpCas9(BB)-2A-Puro(PX459) (Addgene, MA). An HDR template with mutations was cloned into pUC18, and linearized before transfection. Two plasmids with guide sequences and the HDR template carrying either the ERαY537S or ERαD538G mutation were co-transfected into T47D cells. After 3-day selection in 2.5 μg/ml puromycin, followed by 3 weeks of E_2_-free growth, colonies were transferred to a 24-well plate. Clones were genotyped at the DNA and mRNA levels using PCR and restriction enzyme digestion. Sequencing confirmed selected clones. Oligonucleotide sequences and additional details are in Supplementary Methods.

### MTS cell proliferation assay

Cell proliferation assays in 2D culture were performed as described^21^. Briefly, 2,000 cells/well were incubated with treatment for 4 days with one medium change after 2 days. After 4 days, 20 μl of CellTiter 96 Aqueous One Solution (Promega, WI) was added to each well and absorbance measured.

### Quantitative proliferation assays in 3D culture

Quantitative assays of colonies in soft agar were as in ref. 25 with minor modifications. Briefly, 2,000 cells in 60 μl 0.4% agar were plated in 96-well plates above 50 μl 0.6% agar. Cells were grown for 5 days with one medium change, treated with AlamarBlue (Fisher, NH) and fluorescence measured.

### Quantitative reverse transcriptase-PCR (qRT-PCR)

qRT-PCR was as described^63^.

### Protein synthesis

Protein synthesis was quantitated by measuring incorporation of ^35^S-Methionine into newly synthesized protein^21^.

## Additional Information

**How to cite this article**: Mao, C. *et al*. Antiestrogen Resistant Cell Lines Expressing Estrogen Receptor α Mutations Upregulate the Unfolded Protein Response and are Killed by BHPI. *Sci. Rep.* **6**, 34753; doi: 10.1038/srep34753 (2016).

## Supplementary Material

Supplementary Information
