# Supplementary material for: Antiestrogen Resistant Cell Lines Expressing Estrogen Receptor α Mutations Upregulate the Unfolded Protein Response and are Killed by BHPI
Source: Sci Rep. 2016 Oct 7;6:34753. doi: 10.1038/srep34753 (PMC5054422; doi:10.1038/srep34753)
Supplement: Supplementary Information [file srep34753-s1.pdf]

**Supplementary Information**

# Antiestrogen Resistant Cell Lines Expressing Estrogen Receptor $\alpha$ Mutations Upregulate the Unfolded Protein Response and are Killed by BHPI

Chengjian Mao, Mara Livezey, Ji Eun Kim and David J. Shapiro*
Department of Biochemistry, University of Illinois at Urbana-Champaign, Urbana, IL 61801

## Supplementary Methods

**Cell Culture, Reagents and Western Blotting.** T47D, ERαY537S and ERαD538G cells were cultured in MEM with 1% penicillin-streptomycin. T47D cells were maintained in 10% fetal bovine serum (FBS). Before experiments, T47D cells were depleted of endogenous estrogens by maintaining them for 4 days in medium supplemented with 10% charcoal dextran (CD)-FBS. ERαY537S and ERαD538G cells were maintained in 10% CD-FBS. For western blots, cells were treated as indicated in 6-well plates. After treatment, cells were washed with 1XPBS and lysed in RIPA buffer. Western blots were performed as described previously.[20] The following antibodies were used: BiP (#3177; Cell Signaling Technology, MA), $p58^{IPK}$ (#2940; Cell Signaling Technology), ERα (#8644, #13258; Cell Signaling Technology), PR (#3153; Cell Signaling Technology) and β-actin (Sigma, MO). Bound antibodies were detected with horseradish peroxidase-conjugated secondary antibodies and chemiluminescent detection with the ECL2 detection kit (Fisher Scientific, MA) and quantitation by PhosphorImager analysis.

**Cloning and Sequencing.** Forward primer 5'-ATTGGTACCGGCAGCAGAGTTGTGGCTAGTGGAG and reverse primer 5'-AAGTGTCGACCAGGGTGCTGGGCCAATTGTAGGAAC (KpnI and SalI sites used for cloning are underlined) were used to amplify the fragment around the ERα exon 8 region using genomic DNA from T47D cells as template. Q5 high-fidelity DNA polymerase with GC-melt was used (New England Biolabs, MA). The PCR fragment was purified by agarose gel electrophoresis, and then sequenced and compared with gene bank data. 3 single nucleotide polymorphisms were found in the intron region. However, these SNPs are not in the guide sequence region, and therefore do not affect our design.

To make the HDR template, 3 DNA fragments including 2 homologous arms, digested with KpnI/AclI (upstream arm) and SpeI/SalI (downstream arm) (Figure 1a), and a synthesized 62 bp AclI-SpeI fragment containing the ERαD538G or ERαY537S mutations were ligated into pUC18 digested with KpnI and SalI. The primers used to generate the upstream homologous arms are 5’-ATTGGTACCGGCAGCAGAGTTGTGGCTAGTGGAG and 5’-GGGCACAACGTTCTTGCACTTCATGCTG. The underlined sequences are KpnI and AclI restriction sites. Primer set 5’-GCGCCCACTAGTCGTGGAGGGGCATCCGTGGAG and 5’-AAGTGTCGACCAGGGTGCTGGGCCAATTGTAGGAAC was used to generate the downstream homologous arms. The underlined sequences are SpeI and SalI sites, respectively. The 62 nucleotide oligos containing Y537S are 5’-CGTTGTGCCCCTCTCTGACCTGCTGCTGGAGATGCTGGACGCACACCGCCTACTTGCGCCCA and 5’-CTAGTGGGCGCATGTAGGCGGTGTGCGTCCAGCATCTCCAGCAGCAGGTCAGAGAGGGGCACAA. To generate the D538G mutant, the following pair of oligos were used, 5’-CGTTGTGCCCCTCTATGGGCTGCTGCTGGAGATGCTGGACGCACACCGCCTACTTGCGCCCA and 5’-CTAGTGGGCGCATGTAGGCGGTGTGCGTCCAGCATCTCCAGCAGCAGCCCATAGAGGGGCACAA. Before ligation, these pairs of oligos were annealed in 10 mM Tris buffer (pH 8.0) with 100 mM NaCl.

Oligo sets containing guide sequence 1 and 2 are 5’-CACCGTAGTGGGCGCATGTAGGCGG and 5’-AAACCCGCCTACATGCGCCCACTAC, and 5’-CACCGCAGCATGAAGTGCAAGAACG and 5’-AAACCGTTCTTGCACTTCATGCTGC, respectively. These sets were annealed and cloned

into the vector pSpCas9(BB)-2A-Puro(PX459) (Addgene, MA) digested with BbsI, as described in the Zhang lab protocol.[46]

**Transfection and Selection.** To deliver the plasmid expressing guide sequences, the Cas9 gene and homology-directed repair template to T47D cells, low toxicity Lipofectamine 3000 (Fisher, NH) was used following the manufacturer's instructions. Briefly, one day before transfection, 1.5 million cells were plated in a 100 mm plate. The next day, 5 µg of plasmid DNA for each guide sequence, and 10 µg of linearized HDR template plasmid containing either the ERαY537S or ERαD538G mutation was co-transfected into each plate. The ratio of DNA to Lipofectamine 3000 was 1:3. The cells were incubated with DNA-Lipofectamine 3000 complex for 24 hours, after which the medium was replaced with normal growth medium. To select transfected cells, after 24 hours for recovery, the cells were maintained in regular growth medium plus 2.5 µg/ml puromycin. Puromycin selection was carried out for 3 days, with the medium changed daily. After selection, the cells were allowed to recover for 1 day in regular growth medium. The cells from each 100 mm plate were then harvested by trypsin-EDTA and split into 4X 100 mm plates in MEM supplemented with 10% CD-FBS and pen-strep, plus 30% conditioned medium. Conditioned medium was collected from wild-type T47D cells; when the T47D cells reached ~30% confluence, standard medium supplemented with 10% CD-FBS was added. After 2 days, the medium was collected and filtered through a 0.2 µM filtration unit. The resulting conditioned medium can be stored up to 2 weeks at 4 °C. The medium was changed every 4 days until colonies were visible to the naked eye. The colonies were washed twice with 1X PBS, and were kept in 1X PBS for picking under a microscope with a p20 micropipette. The picked colonies were digested in 20 µl of trypsin in a 96-well plate and transferred to a 24-well plate. After a

week of growth, half of the cells were split from the 24-well plate to a 12-well plate and another half to a T25 flask. The cells from the 12-well plate were used for genomic DNA preparation using the Blood & Tissue DNeasy kit (Qiagen, CA).

**Genotyping.** The primers for genomic genotyping are located just outside of the HDR template arms, their sequences are: 5'-CATGGCAAGTCTCCAACTTGAGCTG and 5'-AGTCAGCCTGAGTAGTGACAGCAAC. Genomic DNA from each individual clone was used as a template to amplify a fragment that covers the whole HDR template. To verify insertion of the restriction sites by HDR, the PCR product was digested with AclI or SpeI and bands were visualized by agarose gel electrophoresis followed by staining with ethidium bromide.

To confirm homologous recombination in these positive clones at the mRNA level, total mRNA was prepared. PCR following reverse transcription was carried out to generate an ERα cDNA fragment using a set of ERα-specific primers: 5'-AATCCTCACGCTTAGTAACATA as the RT primer, and 5'-ACAGGGAGCTGGTTCACATGATCAAC and 5'-GAGAGCTGTTACAAAGATTTAGCCTTGG primer set for PCR. The resulting PCR product was then digested with AclI or SpeI to verify HDR at the mRNA level. To confirm the presence of the mutations, the digested or undigested DNA bands were recovered from the agarose gels and sequenced.

## Supplementary Figures

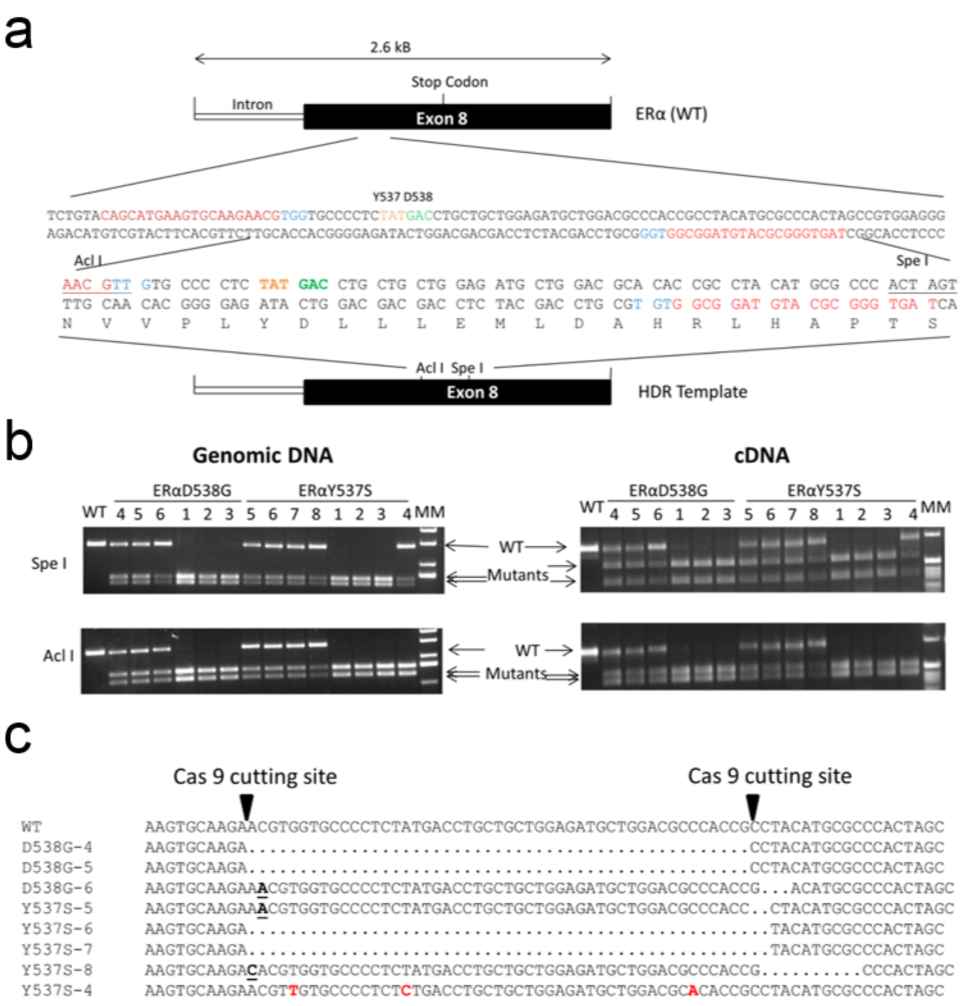


**Supplementary Figure S1.** CRISPR-Cas9 replacement of wild-type ERα with ERαY537S and ERαD538G. (a) Design of the HDR template for gene replacement. The ERα genomic fragment containing part of intron 7 and exon 8 was amplified by PCR, sequenced and compared to database sequences. Blue nucleotides indicate the native or mutated PAM sequence. The single nucleotide changes do not alter the amino acid sequence of ERα or use rare codons. These changes will block cutting of the edited gene by Cas9. Guide sequences are in red. Underlined regions indicate single nt changes to generate AclI or SpeI sites that do not alter the amino acid

sequence. The changes to yield the desired mutations were: ERαY537S: TAT to TCT (orange); ERαD538G: GAC to GGG (green). (b) Genotyping of cell lines: Analysis of genomic DNA and cDNA from clonal cell lines for restriction sites indicating gene replacement. Genomic DNA or cDNA generated from total RNA was used as template for PCR amplification with ERα specific primers. The PCR primers were outside the HDR template or corresponding mRNA region. PCR products from each clone were subjected to AclI or SpeI digestion. Digestion patterns after agarose gel electrophoresis and staining with ethidium bromide are shown for 14 randomly chosen clones, 8 from Y537S and 6 from D538G. (c) Identification of insertions and deletions (indels) in the non-replaced copy of ERα. The undigested (SpeI and AclI) DNA bands from cell lines in which one copy of wild-type ERα was replaced (single replacement) were purified by -agarose gel electrophoresis and sequenced. For both guide sequences, the Cas9 cleavage site is noted. Inserted nucleotides are underlined and deletions are shown by dots. The sequence of all digested bands are as designed (not shown). In ERαY537S-4 one ERα gene is missing the SpeI site and sequencing confirmed the presence of the ERαY537S mutation and mutation of both PAM sequences. Therefore, we can confirm two distinct and independent events replaced both copies of wild-type ERα. This also suggests a homologous recombination event between the restriction site and the PAM sequence. Since sequencing the undigested SpeI ERαY537S-4 band confirmed the ERαY537S and PAM sequence mutations, one of the two homologous recombination events occurred in the 50 nucleotide region between the Y537S mutation and SpeI site. This is the only recombination event observed within 50 bp of the mutation site among all 50 clones (1 out of 130 possible recombination events [potentially 4 recombination events/double replacement]). Thus, using long 1.2 kb homologous arms likely increased the probability of HDR.

a

**Summary of CRISPR-Cas9 Gene Replacement**

| | Both ERα Copies Replaced | One ERα Copy Replaced | False Positive |
|---|---|---|---|
| **ERαY537S** | 9 | 23 | 14 |
| **ERαD538G** | 6 | 12 | 1 |
| **Total** | 15 | 35 | 15 |

b

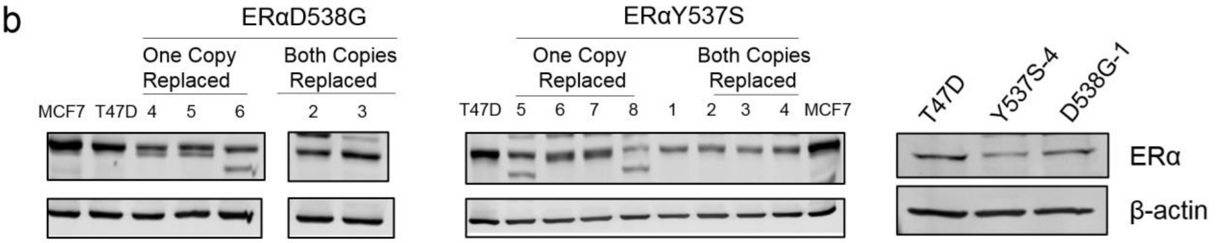


**Supplementary Figure S2.** Summary of CRISPR-Cas9 gene replacement. (a) Table 1 summarizes data for all the clonal cell lines genotyped. Despite the low frequency of HDR for both the ERαY537S and ERαD538G mutations, almost half as many assayed cell lines had both copies of the wild-type ERα replaced with the mutation as cell lines that had one copy replaced. Although we did not observe a substantial growth advantage for the double replacement cell lines in standard cell culture, it remains possible that they exhibited a somewhat larger growth advantage during outgrowth of the clones. The reason for the small number of false positive cell lines in the ERαD538G experiments is unknown. (b) Western blot analysis of ERα expression. To ensure detection of deletions and truncations in the C-terminal region of ERα, we used an antibody recognizing epitopes in the N-terminal region of ERα. The Western blot shows that the pattern of ERα gene expression in the cell lines replacing one copy of the ERα gene matches that predicted from the DNA sequencing. For example: In Supplementary Figure S1c, clones ERαD538G-4 and ERαD538G-5 show a deletion of 17 amino acids and a reading frame shift

after the deletion. This results in a protein, containing 588 amino acids with almost 60 amino acids after a reading frame shift. This protein's size is very close to the size of wild-type ERα as seen by two bands in the western (595 aa). For clones D538G-6, Y537S-5 and Y537S-8, a single nt insertion causes a reading frame shift and premature termination, resulting in truncation of the protein. For these 3 clones, this insertion results in a protein with 537 total amino acids seen as a second, lower molecular weight band than wild-type ERα. For clones Y537S-6 and Y537S-7, there are deletions and reading frame shifts, resulting in a protein containing 579 amino acids. On the gel, the ERα band is broad and blurry, suggesting two bands.

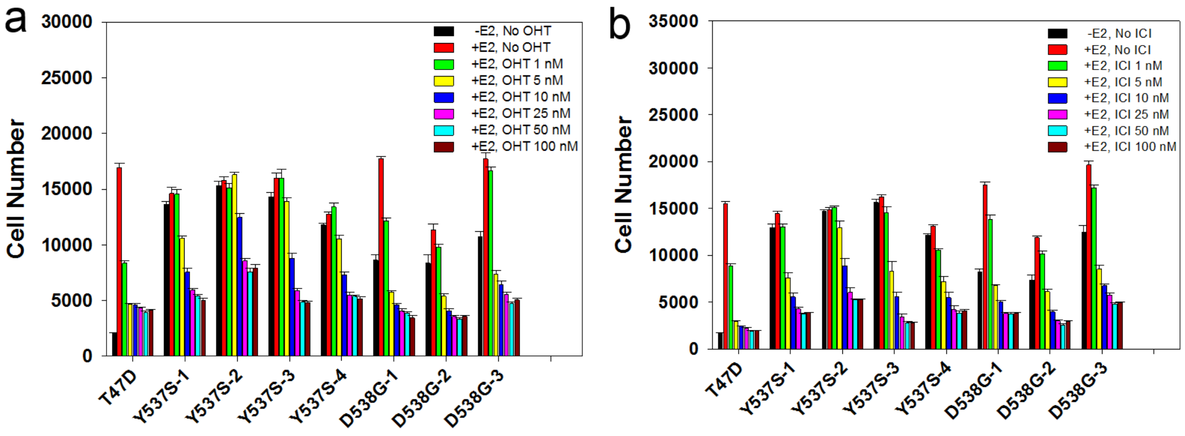


**Supplementary Figure S3.** In standard 2D culture, additional ERαY537S and ERαD538G cells exhibit partial resistance to OHT and ICI. Dose-response studies comparing the effects of increasing concentrations of OHT (a) and ICI (b) on the proliferation of T47D and additional double replacement ERαY537S and ERαD538G cell lines. Data is mean ± SEM (n=8).

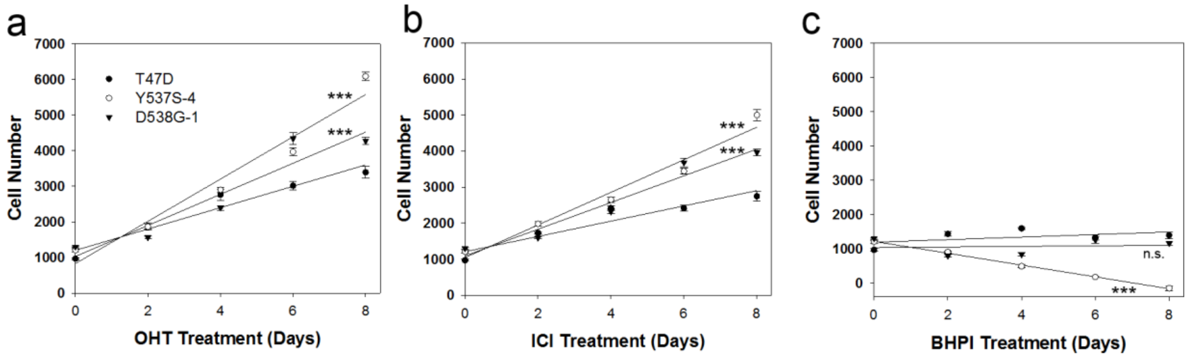


**Supplementary Figure S4.** In longer-term 2D culture, ERαY537S-4 and ERαD538G-1 cells exhibit continued proliferation in the presence of ICI and OHT, but not with BHPI. 1,000 cells were seeded into each well of a 96-well plate. The next day (day 0), treatment was started. Medium with treatment was changed every two days. Data is mean ± SEM (n=8). Slopes of the lines were compared using the free online calculator (http://www.danielsoper.com/statcalc/calculator.aspx?id=103). ***: p<0.001. Statistics were calculated using all collected data (not mean values) comparing the slope of the T47D line to either ERαY537S-4 or ERαD538G-1. Slopes were calculated by linear regression and are as follows- OHT: T47D: 300 ± 22, ERαY537S-4: 593 ± 25, ERαD538G-1: 437 ± 28; ICI: T47D: 214 ± 17, ERαY537S-4: 441 ± 17, ERαD538G-1: 375 ± 18; BHPI: T47D: 37 ± 14, ERαY537S-4: -172 ± 7, ERαD538G-1: 9 ± 15.

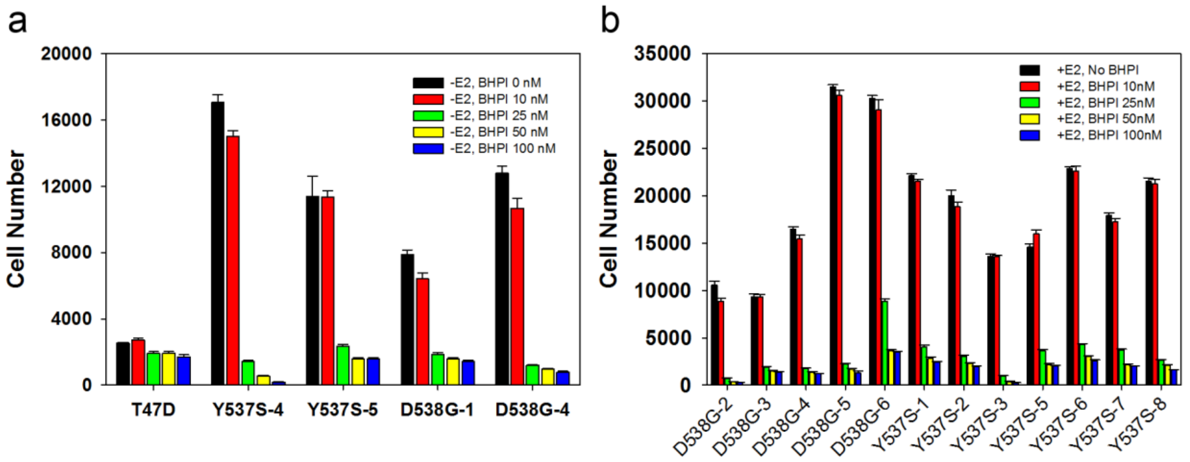


**Supplementary Figure S5.** BHPI blocks proliferation and often kills ERαY537S and ERαD538G cells with or without added $E_2$. (a) Dose-response studies showing 25 nM BHPI is effective in inhibiting proliferation of ERαY537S and ERαD538G cells in the absence of $E_2$. (b) Dose-response studies showing all double and single gene replacement ERαY537S and ERαD538G cell lines are responsive to BHPI in the presence of $E_2$. Independent of the presence of ERα deletions in the non-replaced copy of the single replacement cell lines, or of the formation of ERα heterodimers, BHPI retains full effectiveness and blocks proliferation of every ERα mutant cell line characterized. Data is mean ± SEM (n=8).

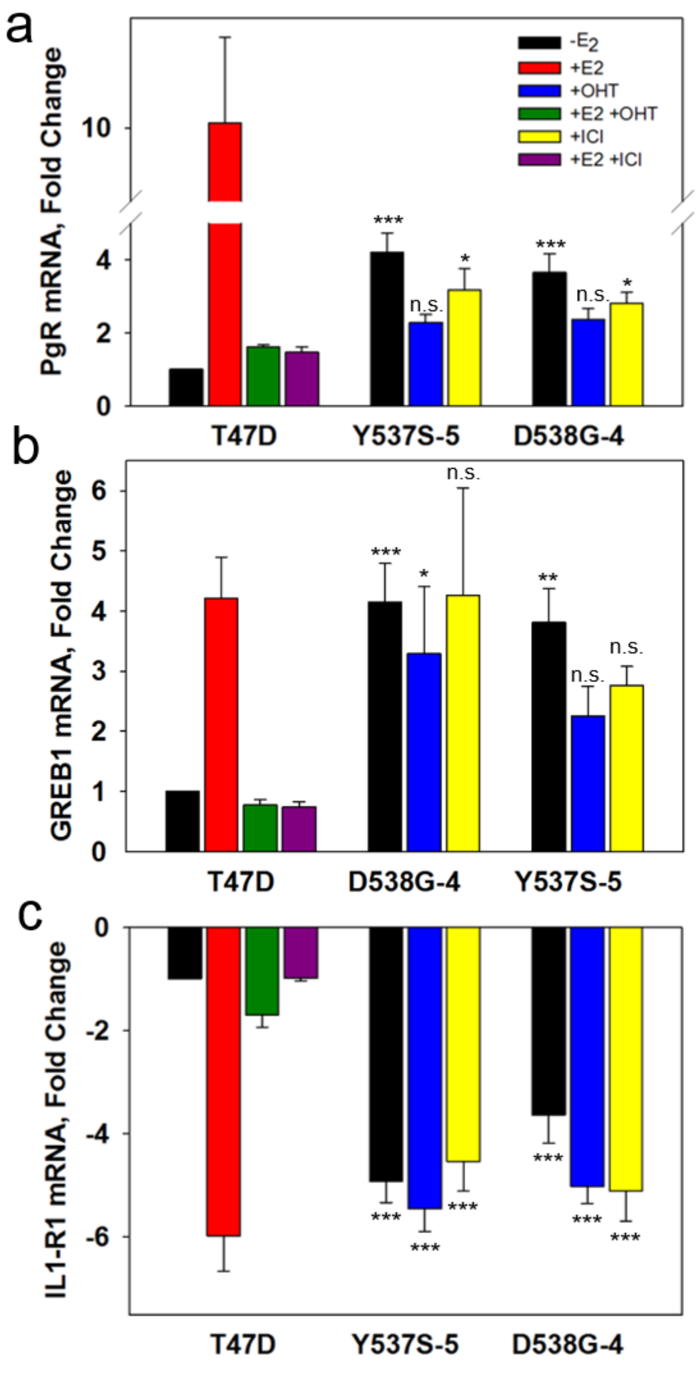


**Supplementary Figure S6.** Expression of ERαY537S-5 and ERαD538G-4 is partially resistant to OHT and ICI. In the ERαY537S-5 and ERαD538G-4 cell lines, one copy of wild type ERα has been replaced with mutant ERα. (a) Levels of PgR mRNA

or (b) GREB1 mRNA after 4 hr treatment and (c) IL1-R1 mRNA after 24 hr treatment in T47D, ERαY537S-5 and ERαD538G-4 cells. . *: $p< 0.05$, **: $p< 0.01$, ***: $p< 0.001$, for each treatment, comparing mutant cell lines to wild type T47D using one-way ANOVA followed by Dunnett's post hoc test. $E_2$ 1 nM; OHT 1 µM; ICI 1 µM; qRT-PCR, mean ± SEM (n=3); -$E_2$ in parental T47D cells set to 1 in (a,b) and -1 in (c).

a

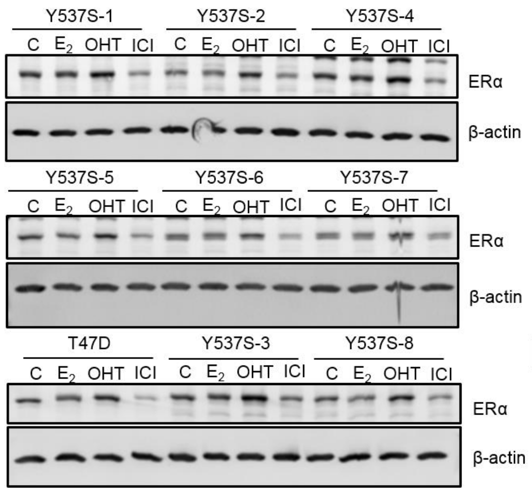


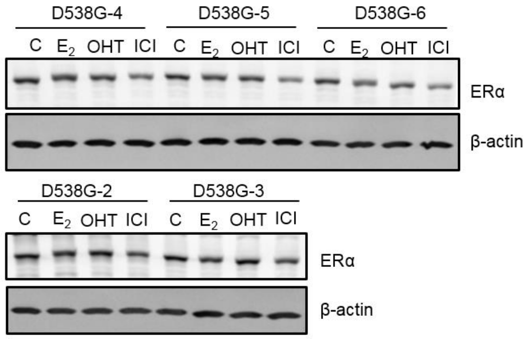


b

**Modulation of Wild Type and Mutant ERα by $E_2$ and Antiestrogens.**

| Fold ERα | Wild type | ERαY537S | ERαD538G |
|---|---|---|---|
| Ctl | 1.00 | 1.00 | 1.00 |
| $E_2$ | 0.74±0.13 | 0.98±0.05 | 0.95±0.03 |
| Z-OHT | 1.92±0.16 | 1.57±0.09 | 0.93±0.05[c] |
| ICI | 0.21±0.02 | 0.50±0.04[c] | 0.51±0.04[c] |

**Supplementary Figure S7.** Effects of ligand on ERα degradation in all single and double replacement ERαY537S and ERαD538G cell lines. (a) Western blot analysis (25 µg/lane) and (b) Densitometry of ERα levels in ligand-treated T47D, ERαY537S and ERαD538G cell lines, within each cell line treatments were compared to vehicle. Data in (b) is mean ± SEM (ERαY537S: n=8 cell lines; ERαD538G: n=6 cell lines; T47D: n=3 experiments); b: $p < 0.01$, c: $p < 0.001$ (by Student's T test), for each treatment, comparing mutant to wild-type ERα in T47D cells.

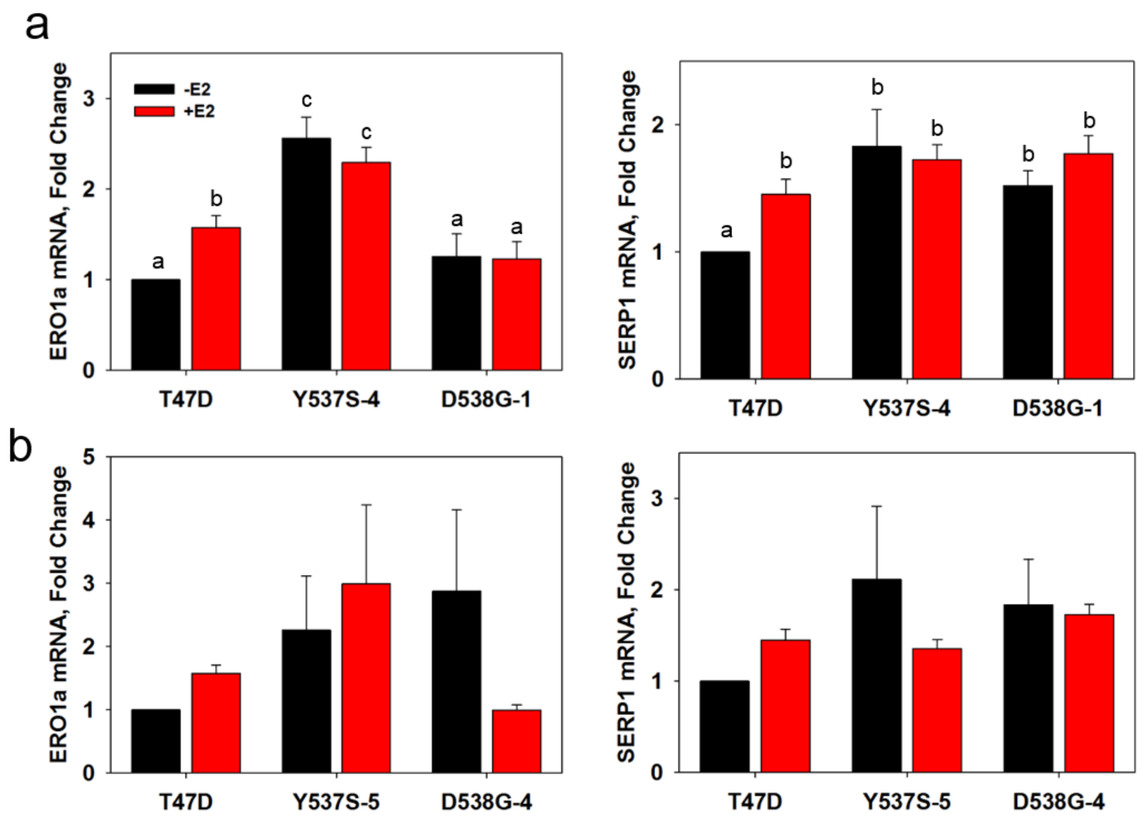


**Supplementary Figure S8.** Expression of UPR marker genes in single and double replacement ERαY537S and ERαD538G cell lines. Levels of ERO1a mRNA and SERP1 mRNA in (a) double replacement and in (b) single replacement cell lines after 4 hr treatment. Different letters indicate a significant difference among groups ($p < 0.05$) using one-way ANOVA followed by Tuckey's post hoc test. $E_2$ 1 nM; qRT-PCR. Data is mean ± SEM (n=3); -$E_2$ in parental T47D cells set to 1 in (a,b) and -1 in (c).
